# Fuzzy Clustering Methods to Identify the Epidemiological Situation and Its Changes in European Countries during COVID-19

**DOI:** 10.3390/e24010014

**Published:** 2021-12-22

**Authors:** Aleksandra Łuczak, Sławomir Kalinowski

**Affiliations:** 1 Department of Finance and Accounting, Faculty of Economics, Poznań University of Life Sciences, ul. Wojska Polskiego 28, 60-637 Poznan, Poland; 2 Institute of Rural and Agricultural Development, Polish Academy of Sciences, ul. Nowy Świat 72, 00-330 Warsaw, Poland

**Keywords:** fuzzy c-means classification method, entropy, COVID-19, epidemic states, Europe

## Abstract

The main research question concerned the identification of changes in the COVID-19 epidemiological situation using fuzzy clustering methods. This research used cross-sectional time series data obtained from the European Centre for Disease Prevention and Control. The identification of country types in terms of epidemiological risk was carried out using the fuzzy c-means clustering method. We also used the entropy index to measure the degree of fuzziness in the classification and evaluate the uncertainty of epidemiological states. The proposed approach allowed us to identify countries’ epidemic states. Moreover, it also made it possible to determine the time of transition from one state to another, as well as to observe fluctuations during changes of state. Three COVID-19 epidemic states were identified in Europe, i.e., stabilisation, destabilisation, and expansion. The methodology is universal and can also be useful for other countries, as well as the research results being important for governments, politicians and other policy-makers working to mitigate the effects of the COVID-19 pandemic.

## 1. Introduction

In the recent past, the coronavirus has become an anomalous part of everyday life worldwide. Moreover, it deepens a sense of insecurity in society and brings confusion due to the lack of standards and rules to fit the new reality. The spread rate of the severe acute respiratory syndrome coronavirus 2 (SARS-CoV-2) that causes COVID-19 and its scale has clearly made everyone aware of how powerful the phenomenon we are dealing with is. In the macro-social dimension, it was associated with disturbances in the economy, i.e., an increase in unemployment, inflation, the budget deficit or a decrease in GDP cf. [1]. On the one hand, people were looking for solutions that would rationalise their everyday life, consisting in a change in everyday functioning, reorganisation of professional life, or changes in the education system. On the other hand, the authorities, started actions aimed at counteracting the unfavourable phenomena in the economy and society. There are many studies and articles on COVID-19 that point to different human behaviour, adaptation to COVID reality, and people’s fear of changes, as well as the process of entering the “new normality” but also negating this phenomenon [2,3,4,5,6,7,8,9,10,11,12,13,14,15,16,17,18,19,20,21,22,23,24,25]. These papers contribute to the literature on the potential healthcare, financial, social, and economic impacts of the COVID-19 pandemic. The importance of this research is highlighted by the European Commission’s “Sustainable Europe 2030”, in which more than half of the ten essential changes needed in the fight the pandemic are economic. These include support for restoring the economy; job protection; financial aid for EU member states; broadening European solidarity, and assisting the economic sectors hit hardest. This goes to show just how vital it is to research the pandemic’s effects.

There are analyses of the situation in countries such as Italy [26], the United States [27,28], United Kingdom [4], Poland [10], Russia [29], Germany [30], China [21,31], Lebanon [23], Kenya [22], Uganda [22,32], Brazil [33], and India [6]. Analyses in these countries reveal various problems, such as rural areas with fewer opportunities for medical services, poorer health and sanitation infrastructure, insufficient social care and numerous problems in managing rural areas, and sometimes endemic poverty. In many countries, such as Canada, the United States, Australia, and Norway, people escaping to the countryside from cities is also a problem, because rural areas have been recognised as places of relative safety [34].

Some studies address issues of studying COVID-19 and its consequences using various mathematical models. Many possible approaches for this modelling can be considered, i.e., non-linear regression, Markov models, differential equation systems (continuous time), and difference equations (discrete time). There are many infectious disease-spread models, such as SIR, SIS, SIRS, SEIR, SEIRD, and SEIHR see e.g., [35,36]. Ivorra et al. [31] developed a mathematical model for the spread of the coronavirus. They proposed the θ-SEIHRD model based on the Be-CoDiS model. Rajaei et al. [37] proposed a different type of nonlinear model for COVID-19. They used a state-estimation-based nonlinear robust control method for state estimation, tracking control, and robustness against uncertainties. Earlier, Sharifi and Moradi [38] proposed a nonlinear epidemiological model of influenza. Shadabfar et al. [28] proposed a probabilistic method to predict the spreading profile of the coronavirus. Their research applied an extended susceptible-exposed-infected-vaccinated-recovered (SEIVR) epidemic model. Moreover, Monte Carlo sampling was used to calculate the exceedance probabilities for three parameters, i.e., the final number of deaths and recovered cases, as well as the maximum number of the infected cases. Moreover, artificial intelligence was applied “in battling against the difficulties the outbreak has caused” [39].

However, there is little research on multiple country analyses together. An interesting example is research by Mahmoudi et al. [40], who studied the situation in the United States, Spain, Italy, Germany, the United Kingdom, France, and Iran. They used a fuzzy clustering technique to compare and cluster the distributions of the spread of COVID-19. It should be noted that, recently, approaches based on soft clustering algorithms have become more popular, having fewer limitations and disadvantages than traditional hard clustering algorithms. Just and Łuczak [41] stated that the “application of classical clustering methods is burdened with some restrictions, which often result in an oversimplification of the actual course of investigated phenomena”. They also added that “the clustering methods based on fuzzy sets provide a much greater amount of information on clustering of objects than classical methods, which only allow the unambiguous assignment each element to one of the clusters”.

Mirkin [42] pointed out that it is possible to “distinguish two overlapping mainstreams potentially leading to bridging the gaps within the clustering discipline. One is related to modeling cluster structures in terms of observed data, and the other is connected with analyzing particular kinds of phenomena”. It is worth adding an observation by Sato-Ilic and Jain [43] that “fuzzy clustering is one method which can capture the uncertainty situation of real data and it is well known that fuzzy clustering can obtain a robust result as compared with conventional hard clustering”.

The statement of these facts leads to reflection on the current situation countries and its changes during the COVID-19 pandemic. Research gaps were identified based on a broad review of the source literature on the classification of objects and studies related to the COVID-19 pandemic. Our goal was to fill a significant research gap in the assessment of the epidemiological situation and its changes in European countries during the coronavirus pandemic on the basis of empirical studies and on this basis to formulate answers to the following research questions:

Q1. What were the typical epidemiological states in Poland and other European countries, from 4 March to 24 June 2020?

Q2. What was the variability of the epidemiological states in the countries analysed from the beginning of the epidemic in Poland until the end of the second stage of the survey?

Q3. Were epidemiological states clearly recognizable in the countries analysed during the given time period?

The main objective of this paper is to identify the epidemic states in the countries investigated from 4 March 2020 (the beginning of the epidemic in Poland) to 24 June 2020 (the first phase—the abolition of most restrictions related to COVID-19 in Poland). Furthermore, the following research hypothesis was formulated: the epidemiological situation in Poland in the period from 4 March to 24 June 2020 was stable compared to other European countries.

To fill the existing research gap, our study identified epidemic states in European countries using the fuzzy *c*-means classification method. The proposed approach not only makes the identification of epidemic states possible, but also provides information on the time of transition from one state to another. Thus, this paper is an important complement to and extension of existing studies on changes in the situation of countries affected by the COVID-19 epidemic. Other authors’ contribution concerned the ability of the entropy of the classification to signal the uncertainty of epidemiological states.

Apart from the introduction, the paper is composed as follows: part 2 presents the methods and data used in the empirical study; part 3 presents the results of the research on the epidemiological situation and its changes in European countries. The final parts (5–7) of the paper present a discussion of the research together with conclusions and recommendations.

## 2. Materials and Methods

The study includes the identification of epidemic states, as well as their changes in European countries from the beginning of the epidemic in Poland (4 March 2020) until the abolition of most restrictions related to COVID-19 in Poland (24 June 2020). The country types were distinguished regarding their epidemiological risk. The cross-sectional time series data from the European Centre for Disease Prevention and Control [44] constitute the empirical basis of the study. Changes in the countries’ epidemic states were identified using the fuzzy *c*-means clustering (FCM) method. FCM “is one of the most classical prototype-based clustering methods” [45]. Yang and Sinaga [46] noted that this method has been “widely extended and applied in various real-world problems, such as pattern recognition, image segmentation, medical diagnostic, economics, cell formation, gene expression, and data mining”.

**Figure 1:**
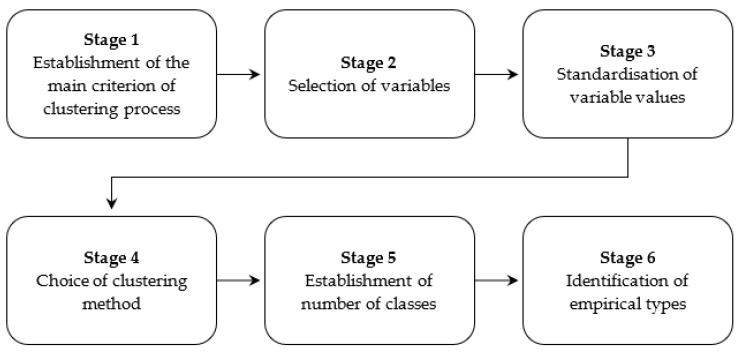
Stages of the clustering process. Source: Own elaboration based on Wysocki [51].

A methodological approach based on clustering methods was proposed (Figure 1). The clustering process consists in the grouping of similar objects [47]. “Clustering mainly aims to partition data into clusters with a maximum similarity in a cluster (homogeneous), as well as a maximum dissimilarity between clusters (heterogeneous)” [48] (p. 297). In other words, Liao [49] (p. 1857) states that “the within-group-object similarity is minimized and the between-group-object dissimilarity is maximized”. It aims to identify relatively homogeneous groups of objects in terms of similar characterising variables. The most frequent clustering methods are the disjoint methods, where each object is assigned only to one class. This indicates that each object is assigned properties of only one type. Such an identification of types is a great simplification of the state of the objects examined, as they frequently possess variables of many types. Methods based on the fuzzy-sets theory help to resolve this issue [50]. This theory was developed to describe highly complex phenomena or poorly defined concepts which cannot be precisely described by the classical mathematical apparatus. In fuzzy-clustering methods, objects may belong to different classes. These methods make it possible to assign objects to all classes with a certain degree of membership.

Prior to the clustering process, it is necessary to establish the main criterion regarding the process (e.g., identification of pandemic states), as well as the objects (e.g., countries) intended for clustering (stage 1). An important stage in the clustering process comprises an appropriate selection of variables (stage 2), which is based on substantive and statistical analyses. The established values of the *K* variables for *n* countries and *T* moments in time are compiled in T⋅N×*K* dimensional data matrix $X^{*}=\left[x_{tik}^{*}\right],$ where $x_{tik}^{*}$ (t=1, 2, …, T;n=1, 2, …, N; k=1, 2, …, K ) is the value of the *k*-th variable for the *i*-th country at time *t*.

Variables describing the objects examined may assume a different nature and their range of maximum and minimum values also varies. The values of variables should be standardised to ensure comparability of data (stage 3). The standardisation, recorded in the form of a matrix $X^{*}$, is conducted according to the formula:

(1) $$x_{tik}=\frac{x_{tik}^{*}-{\bar{x}}_{k}^{*}}{s_{k}^{*}}\;\;\;\;\;\;\;\;(t=1,\;2,\ldots ,\;T;\;i=1,\;\ldots ,\;N;\;k=1,\;\ldots ,\;K),$$

where $x_{tik}$—the standardised value of the *k*-th variable for the *i*-th object at time *t*, $x_{tik}^{*}$—an initial value of the *k*-th variable for the *i*-th object at time *t*, ${\bar{x}}_{k}^{*}$—arithmetical mean of the *k*-th variable, and $s_{k}^{*}$—a standard deviation of the *k*-th variable.

The clustering process is based on the distances between pairs of the multi-variable objects [51,52,53]. The most frequently applied distance measure is the Minkowski distance [52]:

(2) $$d_{ts}={\left\{\sum_{k=1}^{K}{\left|x_{tik}-x_{sik}\right|}^{p}\right\}}^{1/p}\;\;\;\;\;\;\;\;(t,\;s=1,\;\ldots ,\;T;\;i=1,\;\ldots ,\;N)$$

The formula (2) for p=1 comprises a city block (taxicab, Manhattan) distance, which for p=2 is referred to as a Euclidean distance, while for p→∞ as a Chebyshev distance. The application of the city block distance results in cubic clustering, while spherical clustering is identified for Euclidean distances. It should be emphasised that the Minkowski distance is employed to study the similarity of objects with regard to the level of the variable values. 

Moreover, it is necessary to add that it is not possible to indicate a universal clustering method. All methods involve a limitation related to the interpretation of the results obtained, which decreases with the number of objects classified. The most common clustering methods include the *k*-means method and its rarely used fuzzy *c*-means version, both of which were used in this study (stage 4). Knowledge regarding the number of classes, as well as the initial clustering of objects, is required in case of the application of these methods. In the subsequent stages of the clustering process, objects are transferred from one class to another in a way that enables them to minimise the difference from certain class variables (prototypes) within the specific class. The iterative process is repeated until the clustering approaches the assumed level of stability [43,51,54,55].

The clustering of objects requires the number of classes to be determined (stage 5), which may be established by different methods [56,57]. In this paper, the number of classes was determined in two steps. In the initial step, separable clustering was generated using the *k*-means method, then assessed with the Krzanowski–Lai [58] clustering quality index calculated according to the formula

(3) $$KL\left(G\right)=\left|\frac{DIFF\left(G\right)}{DIFF\left(G+1\right)}\right|,\;\;\;\;\;\;\;\;\;KL\left(G\right)\in R$$

where $DIFF\left(G\right)={\left(G-1\right)}^{2/K}trW\left(G-1\right)-G^{2/K}trW\left(G\right)$. When KL(G) reaches the first local maximum for the number of clusters *G* *, the best partition of the population is into *G* * clusters.

In the fifth stage of the clustering, the number of classes adopted was determined by the chosen disjoint clustering method for the identical data matrix. Next, the clustering was conducted using the fuzzy *c*-means method [59,60,61]. The problem of fuzzy clustering was presented as a non-linear issue of mathematical programming [59,60,61]:

(4) $$\mathrm{Minimise}\;\;\;\;\;\;\;\;J_{m}\left(U,C,X\right)=\sum_{t=1}^{T}\sum_{i=1}^{N}\sum_{g=1}^{G}u_{tig}^{m}\sum_{k=1}^{K}{\left(x_{tik}-c_{gk}\right)}^{2}$$

Subject to:

(5) $$\sum_{g=1}^{G}u_{tig}=1\;\;\;\;\;\;(t=1,\;\ldots ,\;T;\;i=1,\;\ldots ,N)$$

(6) $$\sum_{t=1}^{T}\sum_{i=1}^{N}u_{tig}>0\;\;\;\;\;\;\;\;\;\;\;\;(g=1,\;\ldots ,\;G),$$

(7) $$u_{tig}\geq 0\;\;\;\;\;(t=1,\;\ldots ,T;\;i=1,\;\ldots ,N;\;g=1,\;\ldots ,\;G),$$

where *T*—the number of moments in time (e.g., days), *N*—the number of objects (e.g., countries), *G*—the number of fuzzy classes, *K*—the number of variables, *m*—the parameter which regulates the degree of fuzziness of the clustering process, $U=\left[u_{tig}\right]$ − (T·N×G), a dimensional matrix of the degrees of membership of objects belonging to fuzzy classes, $C=\left[c_{gk}\right]$ − (G×K), a dimensional matrix of the centroids (centres of gravity) of classes, and $X=\left[x_{tik}\right]$ − (T·N×K), a dimensional data matrix, where $x_{tik}$ represents the standardised value of the *k*-th variable in the *i*-th object at time *t*. 

As a result of the fuzzy clustering process, each object (e.g., a country at a given moment of time) is classified into each class (epidemic states) with a certain degree of membership, that is, a number between 0 and 1. Additionally, the sum of degrees of membership for each object equals one. The degree of membership determines the strength with which a given object belongs to a particular class (epidemic states). The higher the degree of membership, the more strongly the object is characterised by the variables of a given state. Fuzzy clustering methods provide more information on the clustering of objects than classical methods, which only make it possible to unambiguously assign each object to one of the classes (states) created. The proposed approach not only allows for the identification of epidemic states but also provides information on the time of transition from one state to another, as well as presenting the opportunity to illustrate the fluctuations occurring when states change.

The next stage of the procedure is to identify epidemic states (stage 6). The identification of states may be divided into the formal and the substantive. Formal identification consists of determining the name, while substantive identification involves descriptive statistics of indicators. It is likewise worth paying attention to the fuzziness degree of the classification—we used the entropy index to measure this and at the same time to assess the uncertainty of epidemiological states. Entropy is a measure of the indeterminacy, chaos, and degree of disorder in a structure. It is greater when the states are more equal, and smaller when one state is more pronounced. The entropy of a fuzzy set [62] is a measure of the total amount of information in the missing fuzzy structure, given by a fuzzy set, to such a state that there is no uncertainty in the classification of the elements. The research used the normalized entropy index see [53,60,63,64]:

(8) $$H_{i}=\frac{1}{T}\sum_{t=1}^{T}\sum_{g=1}^{G}h\left(u_{tig}\right)\left(i=1,\;\ldots ,N\right)$$

where:

(9) $$h\left(u_{tig}\right)=\{\begin{array}{c} -u_{tig}\log_{a}u_{tig}\;\;\;\;\;\;\;\;\mathrm{for}\;u_{tig}>0\\ 0\;\;\;\;\;\;\;\;\;\;\;\;\;\;\;\;\;\;\;\;\;\;\;\;\;\;\;\;\mathrm{for}\;u_{tig}=0 \end{array}$$

$u_{tig}$ is the degree of membership of the *i*-th object (country) at time *t* belonging to *g*-th fuzzy class, and a∈(1,∞ ), but usually a=G. Then, this index ranges from 0 to 1. The lower the entropy value, the lower the hesitancy of the states in the period analyzed. In other words, the lower the entropy, the more pronounced is one state, while the higher the entropy, the higher the uncertainty states.

We also determine the changes in the epidemiological states using the daily entropy index:

(10) $$H_{ti}=\overset{G}{\underset{g=1}{\sum }}h\left(u_{tig}\right)$$

Equation (10) “represents Shannon’s measure of statistical uncertainty” [65]. The daily entropy is computed similarly to the normalized entropy index, but aggregating per day. The greater $H_{ti}$, the greater the uncertainty of fuzzy classification; the greater the fuzziness, and the greater the uncertainty in the identification of epidemiological states. It is worth noting that in two extreme cases, if $H_{ti}=0$ then there is no uncertainty in the identification of states, and if $H_{ti}=1$ then we identify the most uncertain situation. 

## 3. Results

The examination of the epidemiological situation, as well as its changes, initiates the adoption of the main objective of the clustering process, comprising the identification of epidemic states. The study covered the European countries and was based on daily data from 4 March to 24 June 2020. A set of four variables (indicators) was selected to identify the epidemic states in the countries studied, as follows:

- COVID-19 cases per 100,000 population (*x*_1_),
- COVID-19 deaths per 100,000 population (*x*_2_),
- share of COVID-19 deaths in COVID-19 cases (%) (*x*_3_),
- active cases—cumulative number for 14 days of COVID-19 cases per 100,000 (*x*_4_).

A statistical description of the variables was presented in Table 1. On this basis, it may be concluded that the variables selected significantly differentiate the countries analysed. Such a conclusion is indicated by a significant range between the maximum and minimum values, as well as by the analysis of the variation coefficient. The largest diversity of values characterised the *x*_2_ COVID-19 deaths per 100,000 population, in which the average diversity of values of this variable in European countries was 339.94%. In European countries, the coefficient of variation of the *x*_1_ COVID-19 cases per 100,000 population was also high (329.17%). The analysis of the variable values based on positional statistics reveals a slightly lower differentiation in their values.

**Figure 2:**
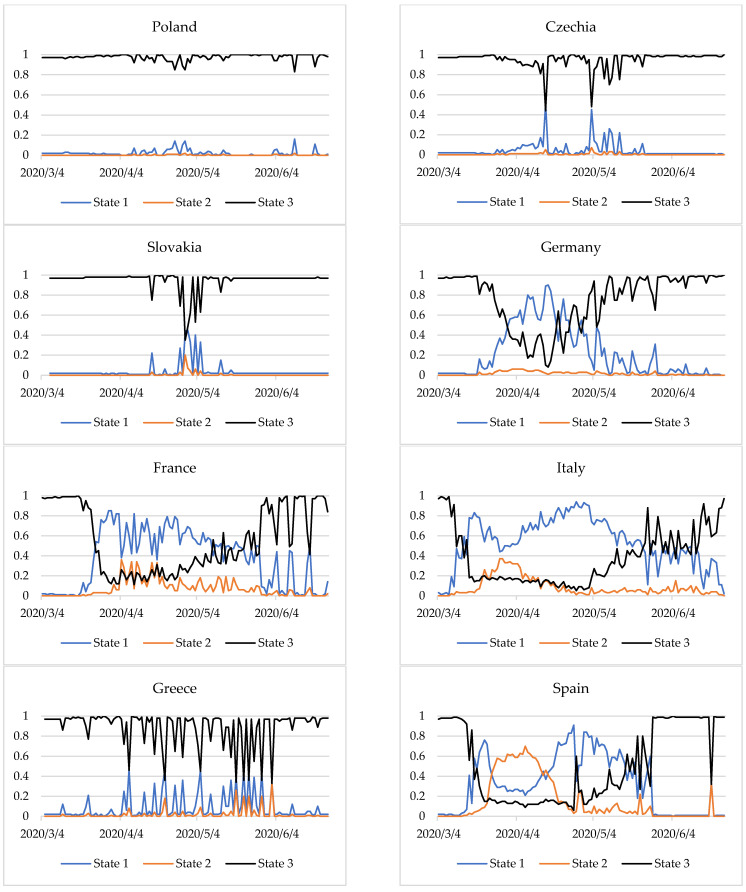
Epidemic states in selected European countries from 4 March to 24 June 2020. Note: The ordinate axis shows the membership degrees of a country for states of the epidemic. Source: own elaboration based on statistical data from [44].

Selected diagnostic variables constitute important information on the epidemiological situation of the countries studied. Initially, sequences of disjoint classifications from 2 to 10 classes were generated using the *k*-means method. The calculations were performed in the R program [66] with the *clusterSim* package [67]. As part of this package, we used a function of the same name, cluster. Sim, for a *k*-means method with the classical standardisation formula for data. The divisions were assessed using the Krzanowski–Lai index, which achieved the first local extremum for three classes. It was therefore assumed that three epidemiological states would be identified in the countries analysed. Subsequently, applying information from the previous research stage, the fuzzy clustering of objects was conducted based on the fuzzy *c*-means method. The calculations were performed in the R program with the *fclust* package [68]. We used the FKM procedure including the fuzzy *c*-means clustering algorithm. The results of the state identification in the countries analysed are presented in Figure 2.

Figure 2 presents the degrees of membership of countries to the three epidemic states in the period examined. The closer the line is to 1, the more identifiable is the state. The change in the membership degrees of countries to specific states indicates a change in the epidemiological state. The method applied makes it possible not only to identify the epidemic states but also provides information on the time of transition from one state to another.

We observed that for Germany, from 20 March 2020 the values of the degrees of membership of the stabilisation state began to decline. This situation lasted until 30 March 2020, with slight fluctuations in the degrees of membership to the stable state. The transition time from stable to destabilisation in Germany was 11 days. On 31 March the degrees of membership to the state of stabilisation and destabilisation were identical at 0.48. From 1 April 2020 Germany entered the state of destabilisation, which finished on 16 April 2020. For the next 19 days, the situation was unstable, and on 5 May (as on March 31), there was no single dominant state and the degrees of membership to the stabilisation and destabilisation states were 0.48. It was only on 6 May that Germany entered the state of stabilisation of the epidemiological situation.

In France, too, the situation began to destabilise around 20 March 2020. The state of destabilisation began after a week. Although this state prevailed until 9 May 2020, sometimes it was only partial (a degree of membership less than 0.5). The situation was ambiguous for the 19 days following 10 May 2020. Only on 29 May 2020 did France enter a state of relative stabilisation of the epidemiological situation. Until the end of the period studied, one can observe a quite regular—about a week apart—sharp decrease in the degree of membership of the state of stabilisation.

In Italy, from 9 March a decrease in the degree of membership of the stabilisation state was observed, lasting about a week. From 16 to 23 May 2020, a state of destabilisation was observed. However, for 23 days from the beginning of May, declining degrees of membership of this state was mostly identified, indicating a potential change in state. For three weeks from 24 May 2020, the situation was unclear. The state of epidemiological stabilisation was mostly identified, but to a large extent it was partial. For three more weeks the situation was not clear. It was only from 14 June that the situation began to stabilise. It should be noted that in Germany, France and Italy the transition from stabilised to destabilised was faster than the other way around.

In Spain, the situation was more complicated. On 16 March 2020 there was a sharp decline in the degree of membership. However, from 12 March a slight decrease in the values of membership degrees was already observed. After about a week, Spain went into a destabilised state. After another week, the expansion of the epidemiological situation already dominated and was identified until 13 April 2020. From 14 April a partial state of destabilisation began to manifest itself, which after a week was already quite intense (membership degrees above 0.7). After another week (27 April 2020), there was a one-day breakdown, followed by a state of destabilisation for the next 19 days. From 17 May 2020 the situation began to stabilise for nine days. This was clear until the end of the period analysed, excluding 16 June 2020, where the degrees of membership to the states were similar (approximately 0.3).

Some countries displayed a stable state throughout the study period. These include Poland, the Czech Republic, and Slovakia. However, Greece was dominated by one epidemiological state—stabilization, but sometimes it was only partial (degrees of membership less than 0.5). This was especially visible in two-week intervals, from 7 to 21 April, from 5 to 19 May and from 22 May to 2 June at intervals of two to four days.

**Figure 3:**
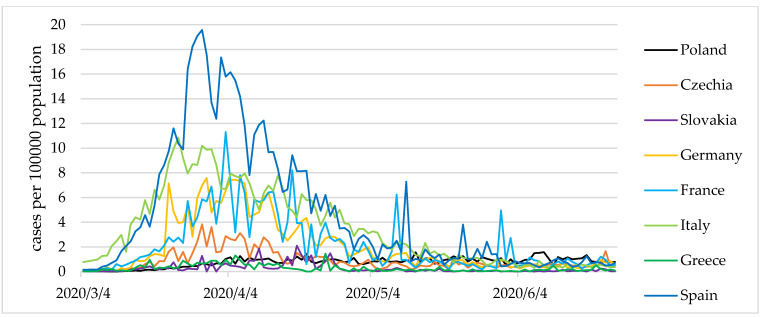
COVID-19 cases per 100,000 population in selected European countries from 4 March to 24 June 2020. Source: own elaboration based on statistical data from [44].

**Figure 4:**
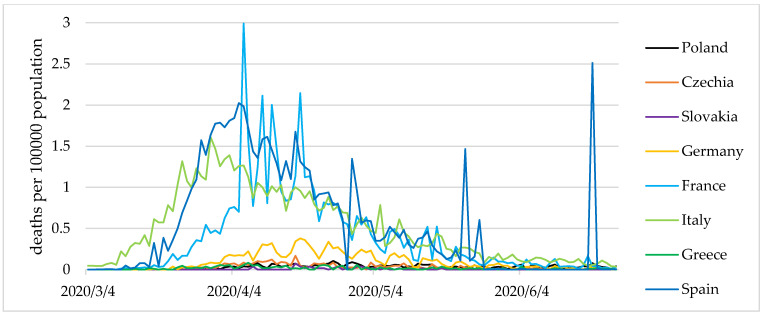
COVID-19 deaths per 100,000 population in selected European countries from 4 March to 24 June 2020. Source: own elaboration based on statistical data from [44].

However, in the latter two countries, a less stable dominant state, as well as small periodic fluctuations were observed. Additionally, Figure 3 and Figure 4 show the values of the COVID-19 cases and present deaths per 100,000 population.

Table 2 presents the affiliation of countries to specific states in three crucial periods: 4 March 2020 (the start of the epidemic in Poland), 15 April 2020, and 24 June 2020. The study identified three main epidemic states in the European countries defined as follows: stabilization, destabilization, and expansion of COVID-19. A state was defined as partial, provided that the highest membership degree of the country was less than 0.5. The degree of membership determines the strength with which a country belongs to a particular epidemic state. The higher the degree of membership, the more strongly the country is characterised by the variables of a given state. The typology of states was conducted using the average values of variables for epidemic states identified in European countries (Table 3).

The analysis of the variable values in the period examined made it possible to identify three epidemic states in Europe. The first state was defined as a total or partial destabilisation. Such a nomenclature was influenced by high values of the indicators. Each of these exceeded the average for Europe as a whole. The number of COVID-19 cases amounted to 5.66 per 100,000 population, with the average for European countries close to 3. It should be noted that there were almost 4 COVID-19 deaths per 1 million population, which represented more than 13.5% of the total COVID-19 cases (%). Simultaneously, the active number of cases within the destabilisation state amounted to nearly 77 COVID-19 cases per 100,000 population. This state constitutes a threat to countries’ economies; however, the level of variables associated with infections and deaths allows for a certain limited functioning of economies.

We called the second state expansion, which constitutes an escalation of the phenomena, noticeable in various intensities. The indicators for state 2 assume significantly worse values than for state 1. The state of expansion was characterised by more than twice as many COVID-19 cases than the state of destabilisation. In the state of coronavirus expansion, the number of active cases increased rapidly, amounting to over 183 COVID-19 cases per 100,000 population. The values of indicators enabled the formulation of a thesis assuming that the situation threatens the country’s stability. They also comprise the basis for social and economic restrictions, resulting in a loss of economic security in the micro- and macro-economic dimensions. Such a state should constitute a premise for a complete or significant closure of the economy to prevent a further uncontrolled expansion of the disease.

In state 3—stabilisation—the values of the indicators were below the European average. The number of COVID-19 cases amounted to 1.57 per 100,000 population, while deaths were at 1 person in over a million. The number of active cases was therefore low (19.64 per 100,000 population), with the European average at 39.8. State 3 does not pose a significant threat to national economies. It appears to constitute a premise for complying with certain hygiene and safety standards, such as the use of masks, hand-washing, and refraining from shaking hands; however, it should not result in a freeze of the national economies. Unfortunately, the absence of recognition of the disease’s effects caused many countries to introduce lockdowns at this level, which resulted in their economic destabilisation.

**Figure 5:**
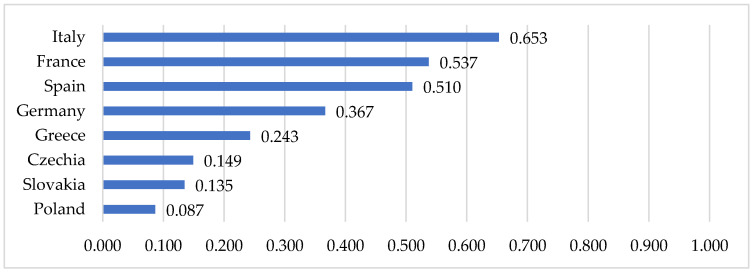
Values of normalised entropy index in selected European countries. Source: own elaboration based on statistical data from [44].

**Figure 6:**
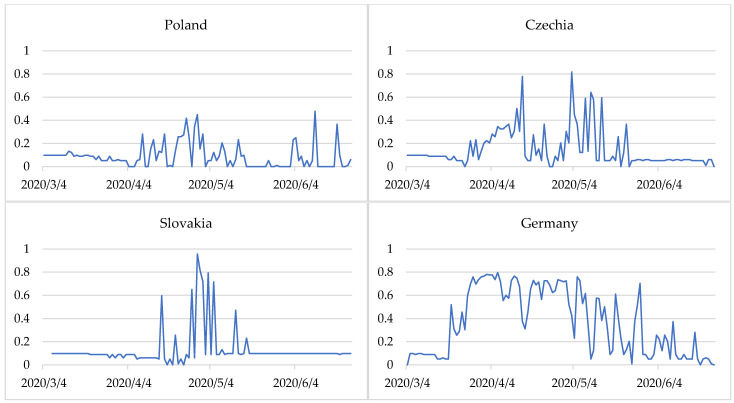

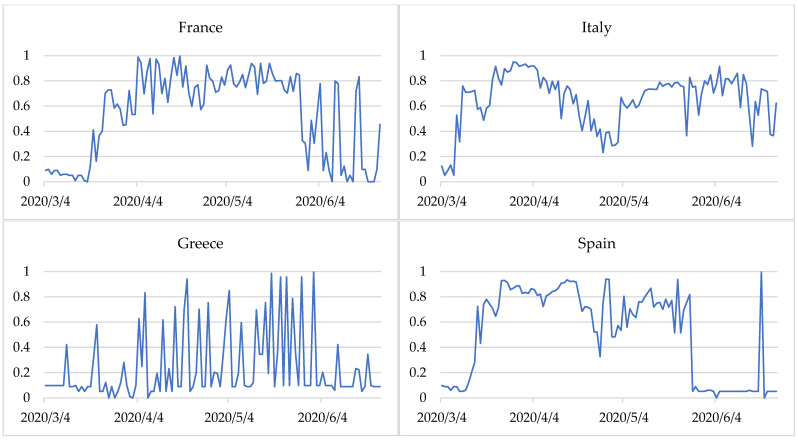
Changes in daily entropy index in selected European countries from 4 March to 24 June 2020. Source: own elaboration based on statistical data from [44].

Figure 5 shows values of the normalised entropy index in selected European countries. A high value of the entropy index was revealed for Italy (0.653). This proves the high uncertainty of the epidemiological situation in the period analysed. A slightly lower value of the entropy index was identified for France (0.537) and Spain (0.510). A very low entropy index value and, at the same time, the most stable epidemiological situation was observed in Poland (0.084). In even greater detail, the uncertainty in the epidemiological situation of countries is shown in the daily entropy index (Figure 6). The results showed that a period of low entropy in countries primarily matches the epidemiological state of stabilisation. This situation was observed especially in Poland (during almost the entire period analysed), Czechia, and Slovakia (from March to until around mid-April and from the end of May to June).

After COVID-19 cases and deaths increased, the entropy was heightened. This shows a destabilisation of the epidemiological situation in Germany, France, Italy, and Spain during almost the entire period considered. In Germany, France, and Italy, during the period studied, there was a transition from a stabilization state (with fewer COVID-19 cases and deaths) to destablilization (with a sharp increase in COVID-19 cases and deaths) and back to a stabilization state (Figure 2). In Spain, the situation was similar, but the state of expansion was also partially identified. Moreover, since the end of May, when the number of COVID-19 cases and deaths was lower, the stabilization state was identified (Figure 2), and the daily entropy index was predominantly at a very low level (Figure 6).

In Greece, the situation was quite different. In this case entropy measure describes the high degree of chaoticity. Although the epidemiological state in the country was mainly identified as stabilization (Figure 2), and the entropy index is quite low—0.243 (Figure 5), the daily entropy shows great variability. This demonstrates the instability of the epidemiological state, despite the country keeping closer to the state of stabilization.

## 4. Discussion

Some studies use models (e.g., the recursive bifurcation model) to describe the infection processes as first- and second-order phase transitions. Such approaches make it possible to show two states, i.e., “the possibility of the population returning to a state with a low level of cases or the epidemic returning” [35]. The advantage of our approach is that more than two states of the epidemic can be revealed. In addition, the proposed fuzzy technique also makes it possible to observe the fluctuations and transition times from one state to another. Variability of states shows the intensity of the process and the hidden diversity in phases of the pandemic.

Moreover, the idea of the proposed fuzzy clustering approach proposed is based on more complex mathematical modelling then in the case of traditional clustering. Important in this approach is the concept of partial membership of a country in more than one class (state). Each country can belong to more than one epidemic state at the same time, but one state a day tends to predominate. The transition from membership to non-membership is gradual. An abrupt transition from one state to another is less common. This relates to the fuzziness of the degree of membership, because “the essence of fuzzy clustering is to consider not only the belonging status to the clusters, but also to consider to what degree do the objects belong to the clusters” [43].

Mahmoudi et al. [40] compared and clustered selected countries using the fuzzy clustering approach. This work describes the distributions of the spread of COVID-19. They also state that “to determine the policies and plans, the study of the relations between the distributions of the spread of this virus in other countries is critical”. Although our research differs, we agree with this statement. It should be emphasised that our research brings a new quality by proposing a fuzzy classification approach to the study of epidemic states in European countries. This approach makes it possible to identify states of pandemic and define the time of transition from one state to another. Our manuscript presents research on the situation of selected European countries, but the research has been conducted for all other countries for which data were available. Our research complements other studies around the world. It outlines the most important background aspects on the epidemiological situation and changes in European countries.

D’Urso et al. [69] used spatial robust fuzzy clustering to identify a clustering structure for the 20 Italian regions according to the main variables related to the COVID-19 pandemic. The exponential distance-based fuzzy *c*-medoids clustering algorithm based on B-splines with a spatial penalty term was applied to the clustering of time series. Although a different fuzzy approach was used and objects at the regional level were studied, three clusters were identified, similar to our study at the European country level. D’Urso et al. [69] obtained “on the entire period almost the same partition”. Our research showed that the variability of epidemic states differed depending on the country. According to our research, in Italy, epidemic states fluctuated even in the initial months of the epidemic.

We should also mention interesting research carried out by Afzal et al. [70]. They used *c*-means and fuzzy *c*-means algorithms for partitioning COVID-19 data. Their results focused mainly on the comparison of the optimum cluster size obtained using both methods. They stated that “the clustering of COVID-19 data from the available data revealed that there were five optimal clusters based on the location and the cases observed so far”, but that “the three main COVID-19 clusters have been identified”. The main number of clusters is therefore in line with our research, although the characteristics of the classes are different and, in our case, more detailed and specific. In our opinion our research allows for a more complex analysis.

Moreover, Ghanbari et al. [71] mentioned that “entropy is related to the missing information on the concrete state of a system and it shows a measure of the disorder of a system”. In that sense we point out that entropy of fuzzy classification can be an effective measure for assessment of an epidemiological situation in a country because it can express a grade of uncertainty (or sometimes stability) of the situation as a single number per day or in a given time period.

It should be emphasised that our research can be extended to include other elements. The research is ongoing and in our opinion, it is interesting to find a connection between studies from the initial pandemic period, which we present in this manuscript, and further studies covering the later phases of the pandemic (e.g., from an annual perspective) and other types of research during the COVID-19 pandemic.

## 5. Conclusions

The study attempted to identify of the COVID-19 epidemic states in European countries. During the period studied, epidemic states and their changes in Poland and other European countries were therefore identified. The fuzzy *c*-means clustering method allowed us to identify countries’ epidemic states. This approach also made it possible to determine the time of transition from one state to another, as well as to observe fluctuations during changes of state. The innovation is the application of fuzzy clusters, which are more appropriate for the characteristics of the variability epidemic states because they avoid a binary split between membership and non-membership.

With this work, we have demonstrated that the entropy analysis of fuzzy classification can contain relevant information concerning the epidemiological states of COVID-19. We demonstrated that the entropy measure of classification can be used to detect the grade of uncertainty in countries’ epidemiological situations. The greater value of the entropy index for a country, the more equal the degrees of membership and, consequently, epidemiological states are less unrecognized (i.e., no one state predominates); the smaller the entropy, the more pronounced is one state. It proved possible to positively verify the paper’s research hypothesis, which stated that the epidemiological situation in Poland from 4 March to 24 June 2020 was stable compared to the other European countries. Three COVID-19 epidemic states were identified in Europe, i.e., stabilisation, destabilisation, and expansion. Our research revealed that one state, defined as stability, dominated the period studied in Poland. The Czech Republic and Slovakia displayed a similar state; however, they had greater fluctuations in the values of the indicators analysed during the same period. Additionally, we also propose a simple way of visualising the countries’ epidemic trajectories in order to enable trend observation and easy comparison. The graphic representation allows for day-by-day monitoring of the epidemic state and its changes.

The message of this research is also that the new public policies currently being introduced have positive but insufficient effects on preventing the spread of COVID-19, and increasing their effectiveness is a must. Hence, the search for new solutions through various types of analysis and research paths concerning the assessment of the epidemiological situation of countries, including changes in their states and dynamics, is very important. Quickly recognising not only states but also the timing of changes from one state to another is extremely important in regard to the authorities’ possible reactions. Producers and consumers alike react to these changes, trying to adapt to them to a certain extent. The accurate identification of any dependencies will, in the future, allow faster responses to threats at an earlier stage.

We believe that research into the epidemiological situation in countries is important in order to understand the trajectory of the COVID-19 pandemic. We believe that scientific analysis and understanding of the various changes in the COVID-19 pandemic can help society better prepare for future outbreaks and support informed decision making in light of societal values. In addition, it should be added that the research results are important for governments, politicians, and other decision-makers who are involved in the process of preventing and reducing the effects of the COVID-19 pandemic.

## 6. Recommendations

The research results can be useful in deepening the understanding of the phenomenon of the COVID-19 pandemic. Above all, the research is significant in illustrating the links between theory and practice in terms of the study of the epidemiological situation in countries. Understanding states of COVID-19 as well as their evolution is of paramount importance for controlling and preventing this disease, and also mitigating the devastating effects of the pandemic. They can therefore be useful in diagnosing and solving real problems, and thus will be useful for decision-makers and politicians involved in the process of developing and implementing COVID-19 prevention policies. Undoubtedly, our research may be useful because it allows us to classify certain groups of countries, to which aid as well as tools for counteracting unfavourable circumstances within the economy and society can, to a greater extent, be standardised. The research makes it possible to get to know the essence of the phenomenon and, as a result, create strategies to prevent threats from occurring or, at the very least, mitigate their effects in the future.

The research concerned European countries, but the results may also be useful for other countries. It is emphasised that the results of this research are based on the state of the COVID-19 pandemic in European countries during its first months, but we are also convinced that the results of this study can be useful for further research during its future phases.

## Figures and Tables

**Table 1:** Values of the selected descriptive statistics of variables characterising the epidemiological situation in the countries examined from 4 March to 24 June 2020.

Variables	Classical Measures					Positional Measures				
	min	mean	max	*SD*	*CV*	*Q*_1_	*Q*_2_	*Q*_3_	*IQR*	*QCOD*
*x*_1_	0.00	3.04	490.80	10.00	329.17	0.16	0.82	2.65	2.48	88.41
*x*_2_	0.00	0.16	17.42	0.53	339.94	0.00	0.01	0.09	0.09	100.00
*x*_3_	0.00	6.07	400.00	16.27	268.05	0.00	0.72	6.39	6.39	100.00
*x*_4_	0.00	39.80	858.90	74.90	188.20	3.77	12.74	40.78	37.02	83.09

Note: *SD*—standard deviation, *CV*—coefficient of variation (%), *Q*_1_—1st quartile, *Q*_2_—median, *Q*_3_—3rd quartile, *IQR*—interquartile range, *QCOD*—quartile coefficient of dispersion (%). Source: own calculation based on statistical data from [44].

**Table 2:** Pandemic states in European countries in crucial study periods.

Date	States	Types of State ^(1)^	Countries ^(2)^
4 March 2020	1	destabilisation	not identified
	2	expansion	not identified
	3	stabilisation	France (0.98) ^(3)^, Austria (0.97), Belarus (0.97), Belgium (0.97), Croatia (0.97), Czechia (0.97), Denmark (0.97), Estonia (0.97), Finland (0.97), Germany (0.97), Iceland (0.97), Ireland (0.97), Italy (0.97), Netherlands (0.97), Norway (0.97), Poland (0.97), Portugal (0.97), Romania (0.97), Russia (0.97), Spain (0.97), Sweden (0.97), Switzerland (0.97), Ukraine (0.97), United Kingdom (0.97), San Marino (0.84)
15 April 2020	1	destabilisation	Germany (0.89), Netherlands (0.89), Switzerland (0.87), Portugal (0.8), Sweden (0.76), Italy (0.69), France (0.63), Denmark (0.62), Norway (0.59), Czechia (0.51)
		partial destabilisation	United Kingdom (0.48), Iceland (0.46), Luxembourg (0.43)
	2	expansion	Ireland (0.70), San Marino (0.57),
		partial expansion	Belgium (0.49), Spain (0.46)
	3	stabilisation	Armenia (1.00), Kosovo (0.99), Russia (0.99), Slovakia (0.99), Ukraine (0.99), Bosnia and Herzegovina (0.99), Georgia (0.98), Lithuania (0.98), Latvia (0.98), Poland (0.96), Greece (0.94), Liechtenstein (0.94), Finland (0.92), Belarus (0.9), Malta (0.88), Bulgaria (0.87), Albania (0.82), Cyprus (0.81), Romania (0.79), Slovenia (0.79), Croatia (0.77), Moldova (0.77), Montenegro (0.72), Monaco (0.63), Serbia (0.63), Austria (0.62), Hungary (0.61), Estonia (0.54), North Macedonia (0.53),
24 June 2020	1	destabilisation	Moldova (0.65), North Macedonia (0.65), Sweden (0.51), Belarus (0.51)
		partial destabilisation	Ireland (0.48), Russia (0.48), Lithuania (0.47)
	2	expansion	Armenia (0.85)
	3	stabilisation	Belgium (1.00), Czechia (1.00), Denmark (1.00), Germany (1.00), Bulgaria (0.99), Serbia (0.99), Spain (0.99), Albania (0.98), Bosnia and Herzegovina (0.98), Croatia (0.98), Cyprus (0.98), Estonia (0.98), Finland (0.98), Georgia (0.98), Greece (0.98), Iceland (0.98), Luxembourg (0.98), Malta (0.98), Monaco (0.98), Montenegro (0.98), Norway (0.98), Poland (0.98), Switzerland (0.98), Ukraine (0.98), Hungary (0.97), Latvia (0.97), Liechtenstein (0.97), Slovakia (0.97), San Marino (0.96), Netherlands (0.94), Romania (0.93), Austria (0.90), Portugal (0.85), France (0.84), United Kingdom (0.80), Kosovo (0.76), Italy (0.72), Slovenia (0.72)

Note: ^(1)^ A type of state was defined as partial, provided that the highest membership degree of the country to a specific state amounted to less than 0.5. The research also included: Armenia, Kosovo, Georgia and Cyprus. ^(2)^ Countries reporting COVID-19 in a particular period. ^(3)^ The highest membership degree of a country to the specific state. The calculations were performed with the *fclust* package [68] in R. Source: own elaboration based on statistical data from [44].

**Table 3:** The average values of variables for epidemic states identified in European countries (average values for fuzzy classes).

Specification	Variables			
	*x*_1_	*x*_2_	*x*_3_	*x*_4_
State 1	5.66	0.39	13.55	76.73
State 2	13.70	0.67	14.23	183.62
State 3	1.57	0.06	3.65	19.64
Mean	3.04	0.16	6.07	39.80

Source: own elaboration based on statistical data from [44].

## Data Availability

All the data supporting reported results comes from: https://data.europa.eu/euodp/en/data/dataset/covid-19-coronavirus-data (accessed on 11 October 2020).
